# The Wells’ Memorial

**Published:** 1894-09

**Authors:** 


### The Wells' Memorial.

The subject of commemorating the introduction of anæsthesia with nitrous oxide by Dr. Wells, of Hartford, Connecticut, was presented to the recent meeting of the American Dental Association, and received the hearty endorsement of that body.

A committee was sometime ago appointed by those specially interested in the subject, to take into consideration the holding of a memorial meeting in Philadelphia. This committee, after fully considering the subject, decided that it was eminently proper, and indeed a matter of justice that such recognition should be made of this notable event; and it was decided to hold such memorial meeting in Philadelphia on the 11th of December next.

At that meeting it is intended to present a more complete statement of facts in connection with this event than has been presented heretofore. This for the sake of correct history is desirable, and for the credit of the dental profession, and especially as a matter of justice and right to Dr. Horace Wells.

It is to be hoped that every practicing dentist who feels an interest in this subject will make it a point to be present at this meeting, and to do whatever he can to make it a success.

The Connecticut State Dental Association proposes to erect an artistic bronze memorial tablet, with portrait of Dr. Wells and an inscription, upon the spot where an operation under an anæsthetic was performed and the discovery made. This tablet will be placed upon the wall of the building which now occupies the exact site of Dr. Wells' office.

The committee, having this work in charge, suggest it is not merely a local matter, but that many practitioners all over the country would consider it a privilege to be on the lists of dentists contributing towards this unique memorial. The committee have sent out a circular to the profession in the United States with the request that all who wish to have a part in this matter should each make a contribution of one dollar or more. Such contributions should be sent to Dr. James McManus, 32 Pratt Sreet, Hartford, Connecticut, who is the Treasurer.

From the knowledge we have already received, we feel free to commend the meeting of December 11th as not only a memorial occasion, but it will undoubtedly be a memorable one as well, and one to be greatly enjoyed by all who may be present.

